# Staining collagen fibers in pituitary lesions substantially improves the certainty of intraoperative frozen section diagnosis

**DOI:** 10.1007/s11102-026-01763-w

**Published:** 2026-09-25

**Authors:** Ursula Keber, Rima Yeghiazaryan, Axel Pagenstecher

**Affiliations:** Institute of Neuropathology, Philipps-University Marburg and University Hospital Marburg, Baldingerstrasse, 35043 Marburg, Germany

**Keywords:** Pituitary adenoma, Pituitary neuroendocrine tumor (PitNET), Cason trichrome collagen fiber stain, Frozen section, Intraoperative histopathological diagnosis, Diagnostic accuracy

## Abstract

**Purpose:**

Transsphenoidal surgery is the first-line treatment for most pituitary adenomas (PA), also known as pituitary neuroendocrine tumors (PitNET), and a precise intraoperative histopathological evaluation of tissue samples is essential to optimize tumor resection and clinical outcome. However, it still remains a challenge for pathologists to distinguish between PA and pituitary gland. In this study, we evaluated the effect of an additional Cason collagen fiber stain on the accuracy of intraoperative histopathological diagnostics of PAs by visualizing either physiologically retained or pathologically lost lobulation.

**Methods:**

A total of 252 cases were included in this study. In the control group (*n* = 93) cytological smear preparations and H&E-stained frozen sections were evaluated. In the experimental group (*n* = 159), both H&E- and Cason-stained frozen sections as well as smear preparations were examined. The concordance rate of the intraoperative diagnoses with the final diagnoses made on the paraffin material was determined.

**Results:**

When compared with the HE only group, the HE+Cason approach markedly increased the diagnostic accuracy from 87% to 98% (*p* < 0.001), sensitivity from 90% to 98% (*p* < 0.05) and specificity from 40% to 100% (*p* = 0.06), whereas the rate of inconclusive cases was significantly reduced from 11% to 0% (*p* < 0.001).

**Conclusion:**

This study introduces the Cason stain on frozen sections as a rapid, simple and inexpensive diagnostic tool essentially improving certainty in intraoperative histopathological diagnostics of PAs, thus facilitating surgical decision-making and optimizing clinical outcomes.

## Introduction

Pituitary adenomas (PA), also known as pituitary neuroendocrine tumors (PitNET), account for approximately 15% of intracranial tumors and constitute over 90% of pituitary lesions [1]. Transsphenoidal surgery is the first line treatment for most symptomatic sellar lesions [2–4]. However, prognosis is complicated either by higher recurrence rates due to incomplete tumor resection or by hypopituitarism following broad resection [5, 6]. Thus, increasing precision of intraoperative histopathological diagnostics including tumor margin identification may substantially improve the postoperative outcome.

The standard procedure of intraoperative histopathological tissue investigation includes microscopic examination of HE stained frozen sections and cytology of smear preparations [7–10]. However, it still remains a diagnostic challenge to distinguish between PA and normal pituitary gland (PitGland) due to small specimen sizes of 1–2 mm, possible freezing artifacts changing cell morphology, cytologically heterogeneous mixed hormone expressing adenomas mimicking PitGland or abundant monomorphous growth-hormone producing cells in the lateral part of pituitary gland mimicking PA. The difficulty in diagnostic evaluation is reflected by reported accuracy rates of intraoperative diagnosis between 83% and 97% [11–13].

A morphological hallmark of PAs is the loss of the physiological lobulation and acinar architecture seen in PitGland. Therefore, the staining of connective tissue septa in cryostat sections for intraoperative diagnosis could substantially increase the diagnostic accuracy. The Cason stain is a trichrome connective tissue stain with a simple and intriguingly rapid staining process comparable to the H&E stain, thus qualifying it for the application in an intraoperative setting. Since the collagen fibers in the frozen section are stained dark blue, the septa in normal pituitary gland tissue are clearly visible. Accordingly, the loss of lobulated and acinar architecture in a PA containing only blue stained blood vessels and residual fibers is easily recognized.

In this study, we evaluate the possible benefit of an additional Cason stain of frozen sections on the certainty of intraoperative neuropathological diagnostics of PAs.

## Materials and methods

### Study design

In total, 252 tissue specimens resected between 2006 and 2022 by the Department of Neurosurgery at the University Hospital Marburg were included in this study. Research use of tissue and data collection were approved by the local ethics committee of the Philipps University of Marburg and all procedures were conducted in accordance with the ethical standards of the Declaration of Helsinki. The tissue samples were evaluated with regard to the concordance rates of the original intraoperative diagnoses based on cytological smear preparation and frozen sections with the original final diagnoses based on evaluation of formalin-fixed and paraffin-embedded (FFPE) material. The diagnostic accuracy of two groups with different intraoperative diagnostic approaches was compared: one group of 159 samples was intraoperatively diagnosed using H&E and Cason-stained frozen sections (HE+Cason), whereas a control group of 93 samples before the introduction of Cason stain in our institute included H&E-stained frozen sections only (HE only). For both groups, additional cytological smear preparations of the specimens were prepared. All slides were reviewed by the same neuropathologist highly experienced in the field of PAs (AP).

### Histopathological analysis

8 μm thick frozen sections were prepared from the fresh surgical specimens, and H&E stain was performed according to the standard protocol. In the HE+Cason group, an additional Cason Trichrome stain was prepared. In detail, the frozen sections were fixed in 5% formalin for one minute, then quickly rinsed with tap water, subsequently stained with Cason Trichrome solution for a minute, followed by a brief rinse in distilled water, and after dehydrating in graded alcohol and clearing in xylene, the specimens were mounted in Eukitt^®^ acrylic-resin-based mounting medium (Table 1). This additional procedure takes less than 4 min from native section to mounted slide.

**Table 1:** Protocol for Cason trichrome staining on frozen sections

Ingredients for a 200 ml stock solution	
2 g	Orange G
3 g	Acid fuchsin
1 g	Water–soluble aniline blue
1 g	Phosphotungstic acid
200 ml Distilled water	

Staining process	
Filter staining solution before use	
Fixation of slide in 5% formalin	1 minute
Rinse with tap water	5 second
Incubate in Cason staining solution	1 minute
Rinse with distilled water	5 second
Dehydration through an ascending alcohol series	
Clearing in xylene	
Mounting	

**Fig. 1:**
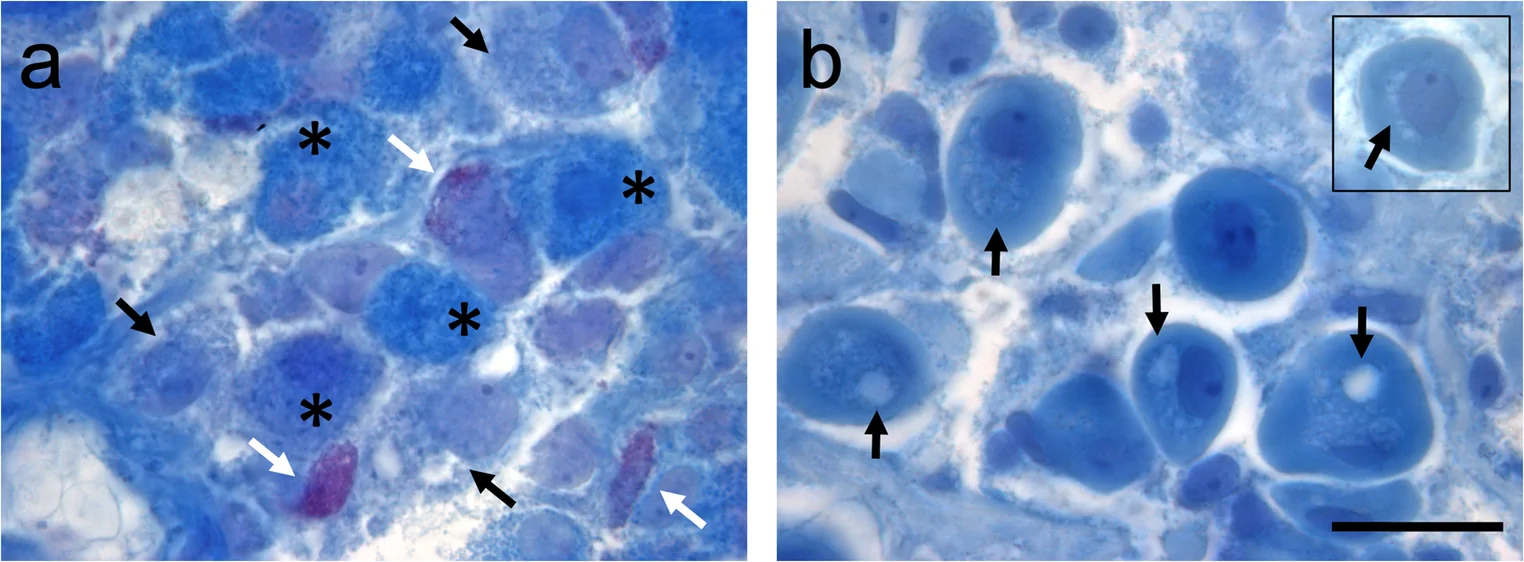
Histology of pituitary gland cell types stained with Cason on frozen sections. (**a**) The three pituitary cell types can be distinguished by a deep blue staining of granules in basophil cells (asterisk), a carmine red staining of granules in acidophilic cells (white arrows) and a greyish staining of granules in chromophobe cells (black arrows). (**b**) In ACTH-producing pituitary cells with Crooke hyalinization, cytoplasm is more homogeneously stained deeply blue with spared hyaline bodies (arrows). Scale bar = 20 μm

The Cason staining solution was prepared as described by Cason [14] in his Mallory-Heidenhain one-step procedure for staining connective tissue: 2 g Orange G, 3 g Acid fuchsin, 1 g water-soluble aniline blue and 1 g phosphotungstic acid were dissolved in 200 ml distilled water (Table 1). The resulting stock solution was filtered and stored at room temperature. For staining a frozen section, the required amount of solution was freshly taken from the stock and filtered again before daily use. This protocol leads to deeply blue stained fibers and to a typical staining of pituitary cell types discussed later (Fig. 1).

Subsequently, the surgical specimens were fixed in formalin and embedded in paraffin (FFPE). 3 μm thick sections were prepared from the FFPE material and used for diagnostic approaches including H&E stain, Gordon-Sweet’s silver impregnation for reticulin fibers and immunohistochemistry for the transcription factors SF-1, Pit-1, TPit/TBX19, the hormones ACTH, prolactin, GH, TSH, LH and FSH as well as the proliferation marker Ki67.

For validation of the Cason stain in frozen sections, an additional frozen section was prepared from several representative tissue samples of the HE+Cason cohort and stained with Gordon Sweet’s silver impregnation. Since the latter stain is time consuming, these sections were not available for the intraoperative neuropathological evaluation of cases.

### Statistical analysis

The accuracy, sensitivity and specificity were calculated and the rate of inconclusive cases was examined. Comparison of these data between the two groups HE+Cason and HE only was assessed using Fisher’s exact test in the software R (version 4.4.0). Graphs were created with GraphPad Prism software (v7).

## Results

The Cason-stained frozen sections of pituitary tissue showed not only a deep blue stain of collagen fibers and violet nuclei, but also a different staining pattern of granules in the pituitary cell types: Eosinophilic cells stained carmine red, basophilic cells deep blue and chromophobe cells grey (Fig. 1). Acidic mucous substances like material in microcysts appeared blue-violet and erythrocytes pale.

**Fig. 2:**
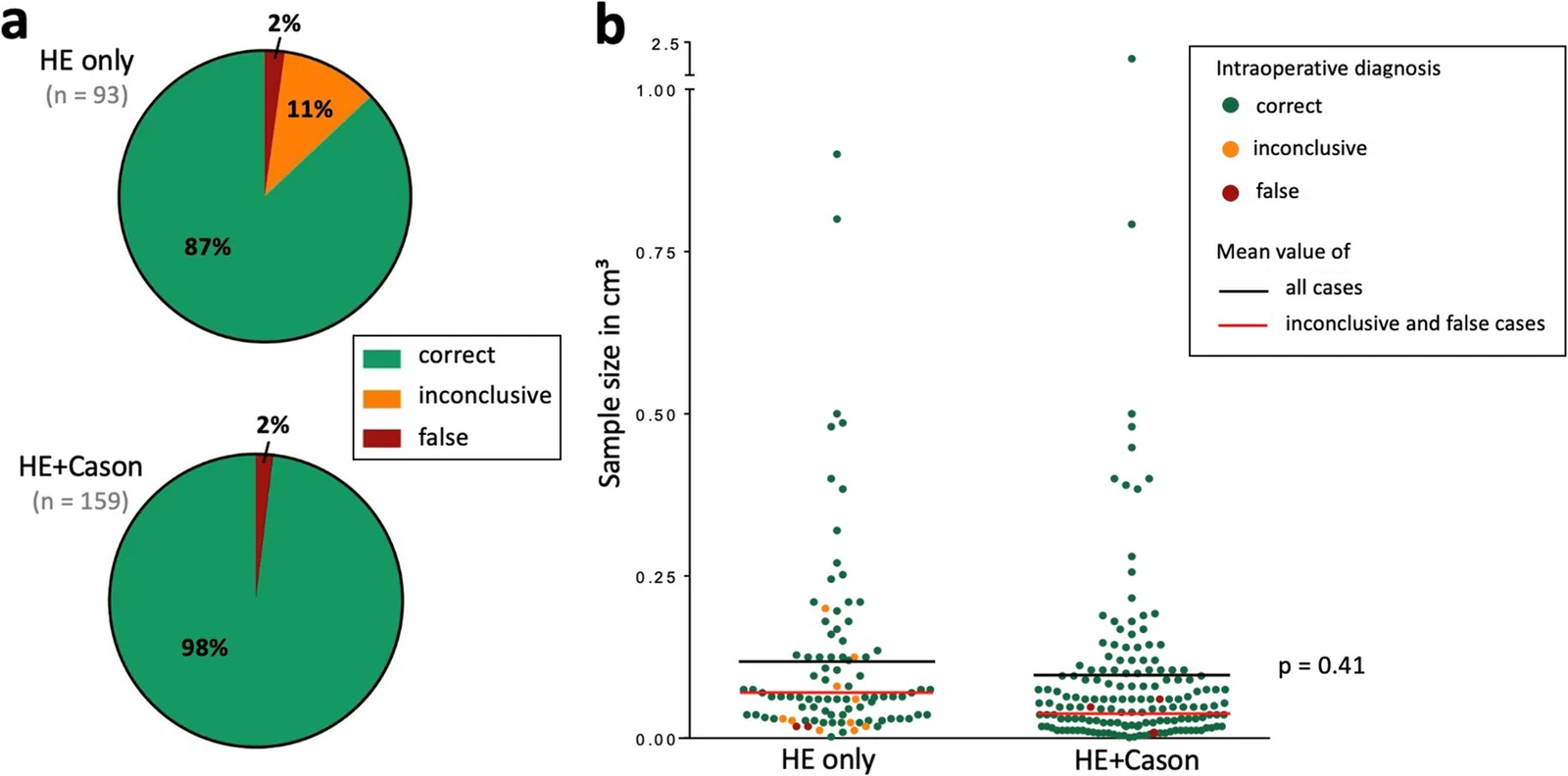
Concordance between intraoperative and final diagnoses (**a**) and sample sizes (**b**). (**a**) The additional use of the Cason staining strikingly eliminated the high percentage of inconclusive cases (orange) and improved diagnostic certainty. (**b**) While the mean value of sample sizes was similar in both groups (black bar), tissue samples with inconclusive (orange dot) or false (red dot) intraoperative diagnoses were on average smaller in the HE+Cason group compared to those in the HE only group (red bar)

In the HE only group, 81/93 intraoperative diagnoses were concordant with the final diagnoses on FFPE material (accuracy 87.1%, Table 2). In detail, 79/88 PAs (sensitivity 89.8%) and 2/5 PitGlands (specificity 40%) were correctly diagnosed on frozen sections. Eight of the nine cases which could not be diagnosed as PA and two of three samples failing the diagnosis of PitGland were intraoperatively described as inconclusive regarding the distinction between PitGland and PA (10.8% inconclusive cases, Fig. 2a). One case with the final diagnosis of gonadotroph PA was intraoperatively misinterpreted as PitGland. Another case with the final diagnosis of PitGland was intraoperatively mistaken as PA with the description of ‘infiltrative’ PA. The nine inconclusively or falsely diagnosed PAs consisted of five gonadotroph, one somatotroph, one lactosomatotroph, one thyrotroph and one corticotroph PA.

**Table 2:** Diagnostic accuracy of intraoperative frozen section stainings

	HE only (n)	HE+Cason (n)	p-value
Sensitivity	89.8 (88)	98.0 (153)	*<0.05*
Specificity	40.0 (5)	100 (6)	*0.06*
Accuracy	87.1 (93)	98.1 (159)	*<0.001*
Inconclusive rate	10.8 (93)	0.0 (159)	*<0.001 *

Date are presented in %, P-values were calculated with the Fisher’s exact test

**Fig. 3:**
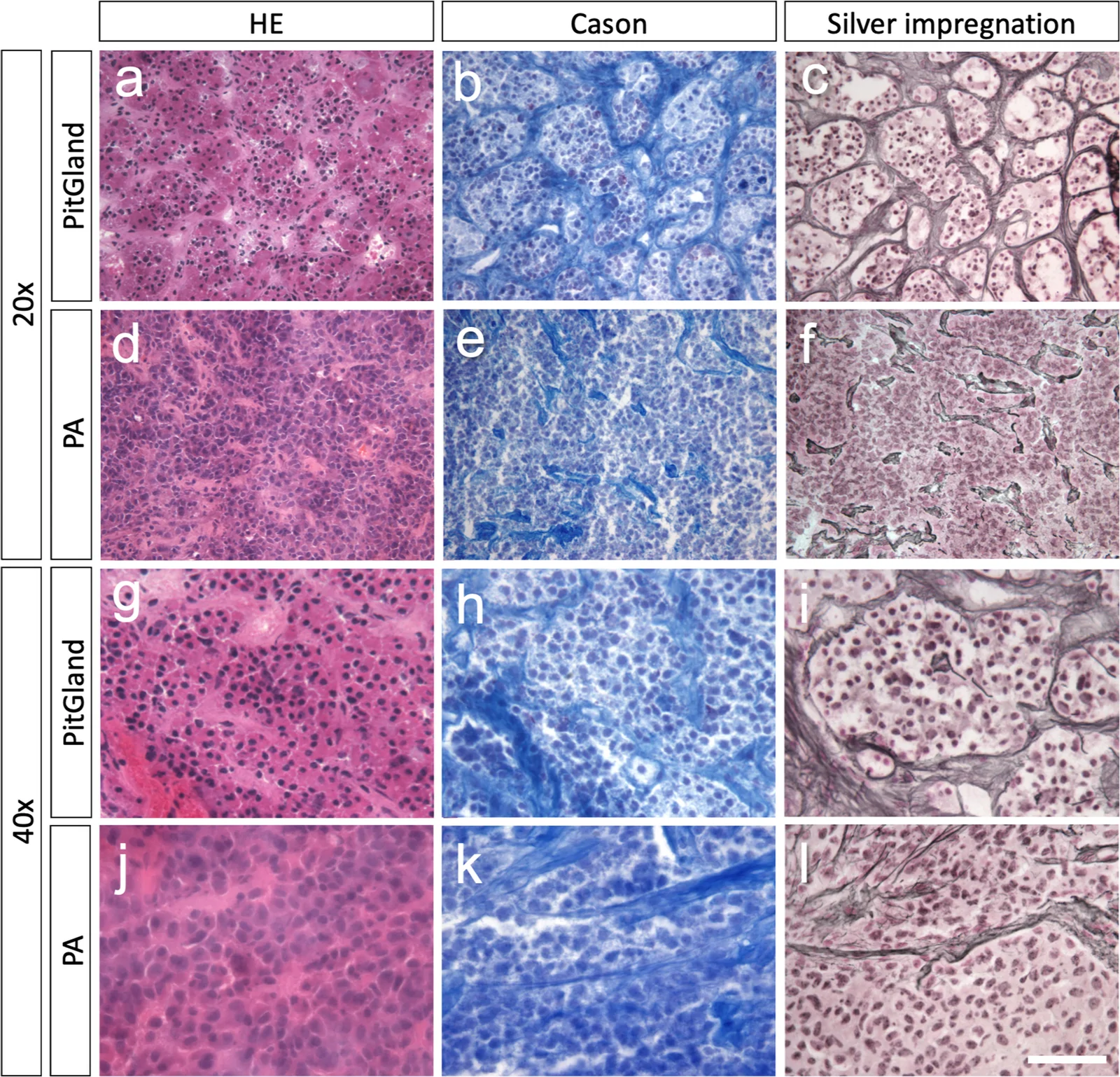
Histomorphology of intraoperatively diagnosed pituitary gland (PitGland) and pituitary adenoma (PA). Frozen sections were stained with HE (a, d, g, j), Cason (b, e, h, k) and Gordon Sweet‘s silver impregnation (c, f, i, l). In the cason staining, septae of PitGland (two cases, a-c and g-i) are stained deeply blue, similar to fibers shown by silver impregnation, whereas in PA (two cases, d-f and j-l) only vessels and residual fibers become visible, allowing for a clear distinction between these diagnoses. Scale bar a-f = 100 μm, g-l = 50 μm

In the HE+Cason group, 156/159 intraoperative diagnoses were concordant with the final diagnoses on FFPE material (accuracy 98.1%, Table 2). In detail, 150/153 samples were correctly diagnosed as PA (sensitivity 98.0%, Fig. 3g-l) and 6/6 as PitGland (specificity 100%, Fig. 3a-f). Remarkably, no case remained inconclusive (0% inconclusive cases, Fig. 2a). Three cases with the final diagnoses of PA were intraoperatively mistaken as PitGland. Of those, one sample was evaluated as pituitary capsule and showed up to be a fibrosing lactotroph PA following cabergolin treatment. The remaining two samples turned out to be gonadotroph PAs and were intraoperatively described as PitGland with dilated lobuli and as mainly blood but scant PitGland lobuli, respectively.

Sample size did not significantly differ between the HE only and HE+Cason group (0.12 ± 0.15 cm³ vs. 0.10 ± 0.21 cm³, *p* > 0.05; Fig. 2b). While cases with inconclusive or false intraoperative diagnoses tended to have smaller sample sizes in both groups, the mean sample size of these cases was considerably larger in the HE-only group than in the HE+Cason group (0.07 ± 0.07 cm³ vs. 0.04 ± 0.03 cm³).

Together, when compared with the HE group, the addition of the Cason stain markedly increased the sensitivity from 90% to 98% (*p* < 0.05), the specificity from 40% to 100% (*p* = 0.06) and the accuracy from 87% to 98% (*p* < 0.001), whereas the rate of inconclusive cases was significantly reduced from 11% to 0% (*p* < 0.001). Although the specifity rose from 40% to 100%, statistical significance was not reached due to the low number of only 5 and 6 PitGland cases in the HE group and the HE+Cason group, respectively (Table 2).

**Fig. 4:**
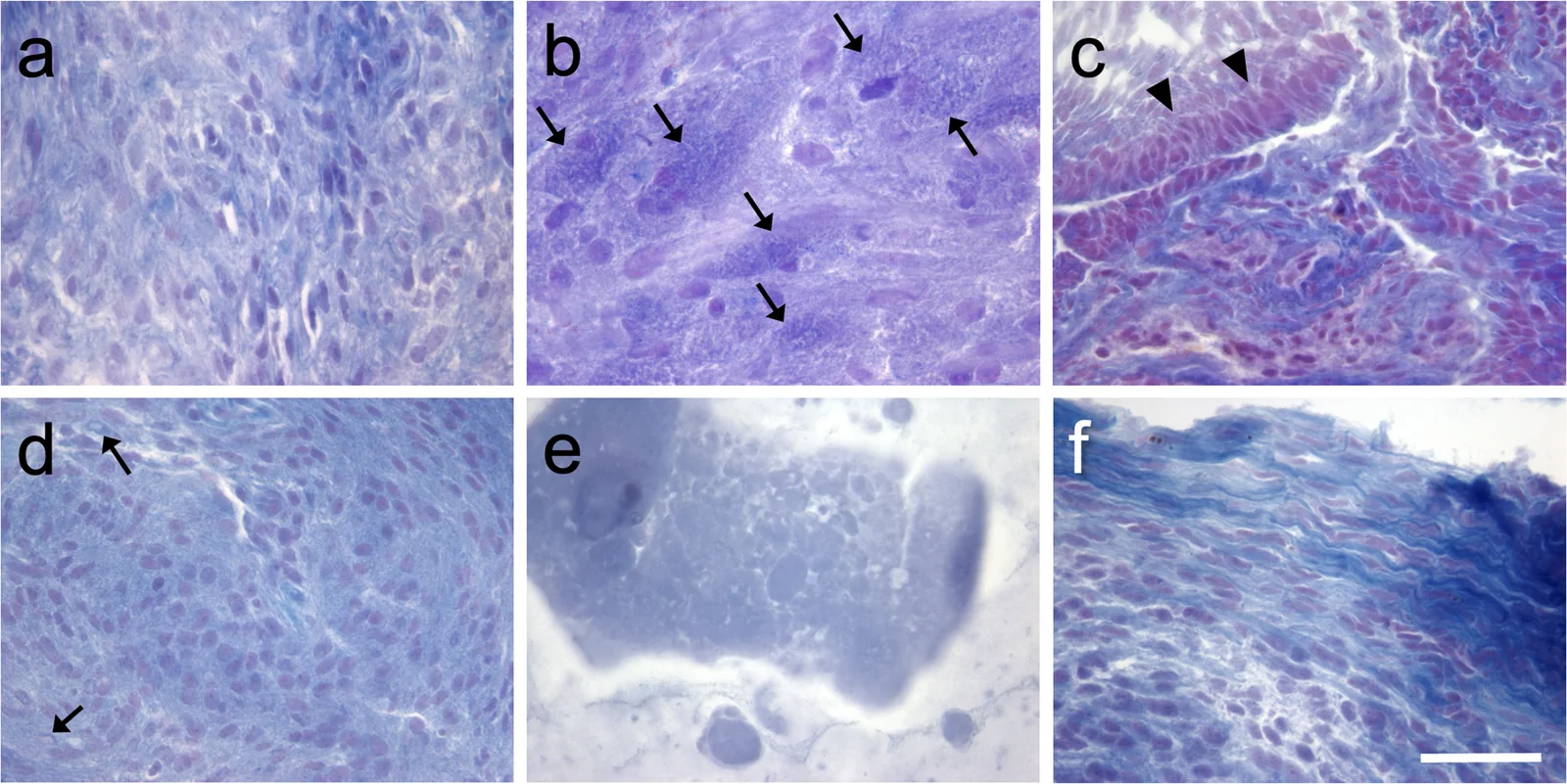
Histomorphology of other sellar processes (**a**-**e**) and fiber containing tumors (**f**) stained with Cason on frozen sections. A low fiber content is not only seen in pituitary adenomas but several differential diagnoses like pituicytomas (**a**), granular cell tumors (**b**, arrows show granulated tumor cells), craniopharyngiomas (**c**, arrowheads show epithelial tumor cells), meningiomas (**d**, arrows show nuclear holes) or cystic material (**e**). Apart from suprasellar lesions, Cason staining may be useful in intraoperative diagnosis of tumors with many fibers like schwannoma (**f**). Scale bar = 100 μm

Apart from PAs, many other suprasellar tumor types showing only few collagen fibers were intraoperatively stained with Cason during the period of this study, i.e. pituicytomas, granular cell tumors, craniopharyngiomas and meningiomas (Fig. 4a-d) as well as cystic material (Fig. 4e). The staining proved also useful for tumors with characteristically many fibers like schwannoma (Fig. 4f).

## Discussion

Here, we present the additional Cason collagen fiber stain of frozen sections as a powerful, rapid and simple tool to significantly increase the certainty in intraoperative histopathological diagnostics of PAs by raising accuracy from 87% to 98%, sensitivity from 90% to 98% and specificity from 40% to 100% compared to the conventional diagnostic setting. The rate of inconclusive cases was strikingly eliminated from 11% to 0% and very small specimens were classified with higher diagnostic confidence. To our knowledge, this is the first introduction of a staining that allows visualization of the pituitary fibrovascular network in less than four minutes from native frozen section to mounted slide. Even more, this is the first study representing the Cason staining on cryosections as a powerful intraoperative diagnostic tool in histopathology in general.

Methodologically, the Cason staining has previously been described for FFPE material with an incubation time of five minutes [14, 15]. Beside the stain of collagen by aniline blue, nuclei and cytoplasms have been stated to stain red by acid fuchsin and erythrocytes orange by Orange G. In the present study, frozen sections of pituitary tissue were stained for only one minute, that sufficed to distinguish the pituitary cell types by different staining pattern of granules (Fig. 1) according to a previous report of Róna and Morvay [16]. This may additionally help to differentiate between PA with only one cell clone and pituitary tissue.

In our experience, at room temperature the Cason stock solution has a shelf life of at least two years, making it also suitable for laboratories that do not encounter pituitary tissue samples on a regular basis. Furthermore, the price of the reagents for the Cason staining solution is less than half that of the H&E staining solutions.

Due to the diagnostic challenge of pituitary lesions in the setting of intraoperative diagnostics, several approaches have been presented in previous studies to facilitate intraoperative pathological decision making. For instance, touch imprint cytology may serve as a useful tool to complement intraoperative neuropathological diagnostics [17–20]. However, as this method lacks information about the tissue architecture and invasion or tumor margins cannot be evaluated, it should be seen only as a supplemental tool in addition to slides.

Silver impregnation for reticulin fiber staining marks the fibrovascular network and considerably facilitates the distinction of lobulated PitGland from PAs or other tumors that lack the physiological lobulation of PitGland [21, 22]. However, the complexity and time requirement of this staining process taking between ten to about 20 min depending on protocol are major drawbacks of this method. Another special staining with orange G-hematoxylin led to a diagnostic accuracy of ‘above 90%’ without reporting detailed results [23].

The fluorescent markers Ricinus communis agglutinin 120 lectin and propidium iodide have been proposed as a helpful diagnostic tool, though no diagnostic concordance rates were evaluated and a fluorescent microscope is required [24]. Similarly, multiphoton microscopy and confocal reflectance microscopy are relatively fast tools that provide promising images of PAs vs. PitGland [25, 26], but both techniques require confocal microscopes with imaging analysis systems. Additionally, there is only little experience for the use of multiphoton microscopy on frozen sections and confocal reflectance microscopy is performed with the same tissue sample before frozen sections can be made, thus leading to an increased time interval until H&E stained slides can be investigated.

Another suggestion of intraoperative tissue ACTH concentration measurements is restricted to patients with Cushing disease and may be falsely positive in case of resected areas rich of ACTH producing pituitary cells or pituitary hyperplasia, as demonstrated by the low specificity of 71% using this parameter [27].

Taken together, none of these methods for frozen sections has found its way into routine diagnostic use because of major drawbacks such as time-consuming protocols, need of expensive specialized equipment or low specificity and sensitivity. In contrast, our present study demonstrates that the Cason stain provides both a high diagnostic certainty as well as a simple and rapid protocol with no special equipment but a single staining solution that allows its broad application in diagnostic laboratories.

The addition of a Cason-stained frozen section to the intraoperative diagnostic routine significantly increases confidence and reduces ambiguity of the neuropathologist as is reflected by the extinction of inconclusive cases in the series reported here. The precise intraoperative diagnosis is critical for the neurosurgeon, who can make informed decisions based on accurate intraoperative guidance. This minimizes post-surgical complications such as hypopituitarism due to inadvertent resection of PitGland or higher recurrence rates and morbidity due to incomplete tumor resection.

Apart from suprasellar tumors, the Cason stain on cryostat sections may also serve as a supporting tool in the intraoperative pathology in general. On the one hand, it may help differentiate other tumor types with high fiber content, i.e. sarcomatoid tumors or nerve sheath tumors such as schwannomas (Fig. 4f). On the other hand, it could be used to evaluate tumor invasion into adjacent local tissue.

A limitation of this study is its retrospective design with a historical control group. Although the investigator was the same person for evaluation of cases in both HE only and HE+Cason group, the utility of a historical control group may be confounded by the experience gained by the neuropathologist during the period of this study.

Additionally, this study focused only on the discrimination between the pituitary gland and PAs. Caution should be given not to misinterpret any pituitary lesion with low collagen fiber content in tissue samples as PA. Other suprasellar processes such as pituicytomas (Fig. 4a), spindle cell oncocytomas, granular cell tumors (Fig. 4b), craniopharyngiomas (Fig. 4c), glial or glioneuronal tumors, germinomas, meningiomas (Fig. 4d), lymphomas or cystic (Fig. 4e) and inflammatory lesions must be taken into consideration and ruled out. In the case of fiber-rich tissue samples, such as pituitary capsule, a pre-treated lactotroph PA or tumor-invaded fibrous tissue, the Cason staining highlights fibers, but it still cannot distinguish their origin. Therefore, evaluation of the overall morphology remains crucial.

In conclusion, our study introduces the Cason stain of frozen sections as a rapid, simple and inexpensive diagnostic tool improving certainty in intraoperative histopathological diagnostics of PAs, thereby facilitating surgical intraoperative decision making and optimizing clinical outcomes of patients.

## Data Availability

The data that support the findings of this study are based on diagnostic neuropathology reports and are not publicly available due to patient confidentiality and institutional data protection regulations. Access to these data is restricted to authorized personnel of the Institute of Neuropathology, University Hospital Marburg. Data may be made available from the corresponding author upon reasonable request and subject to approval by the Institute of Neuropathology and applicable legal and ethical requirements.
